# Pamphlets and Exchanges

**Published:** 1898-09

**Authors:** 


### PAMPHLETS AND EXCHANGES.

---

Reprints received:

Cataract Operations: Mule's Operation; Capsulotomy; Operation for Pterygium. By L. Webster Fox, A.M., M.D., Philadelphia.

A Homily on Doctor's Fees; The Hippocratic Oath; Early Signs of Conception. By W. Symington Brown, Stoneham, Mass.

The Tuberculin Test in Cervical Adenitis. By Edward O. Otis, M.D., Boston, Mass.

The Newer Preparations of Bismuth. By W. R. Wilcox, M.D., of New York.

The Essential of the Art of Medicine. By J. H. Musser, M.D., Philadelphia.

The Diagnostic Importance of Fever in Late Syphilis. By J. H. Musser, M.D., Philadelphia.

Renal Calculus. By J. H. Musser, M.D., Philadelphia.

The Myocardium. By J. H. Musser, M.D., and J. D. Steele, M.D., Philadelphia.

Diseases of the Lachrymal Passage—Their Causes and Management. By Leartus Connor, A.M., M.D., Detroit.

The Prevention of Diseases now Preying upon the Medical Profession. By Leartus Connor, A.M., M.D., Detroit.

---

*Collier's Weekly* is certainly giving to the public the best pictorial representation of the war that can be found in any weekly or monthly magazine. Besides this, its war comments are exceedingly entertaining. It is certainly the weekly magazine for the home.

---

ONE of the most interesting of our exchanges is the monthly journal *Items of Interest*, published by the Consolidated Dental Manufacturing Company, of New York. It is bright, newsy, and thoroughly up to date. We note in the July number an excellent photographic description of one of Atlanta's best dentists—Dr. T. P. Hinman.

